# An Individualised Nutritional Intervention Concept for Nursing Home Residents with or at Risk of Malnutrition: An *enable* Study

**DOI:** 10.3390/geriatrics6010002

**Published:** 2020-12-26

**Authors:** Johanna Seemer, Eva Kiesswetter, Anne Blawert, Daniela Fleckenstein, Marina Gloning, Stephanie Bader-Mittermaier, Cornel C. Sieber, Susanne Wurm, Dorothee Volkert

**Affiliations:** 1 Institute for Biomedicine of Aging, Friedrich-Alexander-Universität Erlangen-Nürnberg, 90408 Nuremberg, Germany; eva.kiesswetter@fau.de (E.K.); cornel.sieber@fau.de (C.C.S.); dorothee.volkert@fau.de (D.V.); 2 Section Social Medicine and Prevention, Institute of Community Medicine, Universitätsmedizin Greifswald, 17475 Greifswald, Germany; anne.blawert@med.uni-greifswald.de (A.B.); Susanne.Wurm@med.uni-greifswald.de (S.W.); 3 Fraunhofer-Institute for Process Engineering and Packaging, 85354 Freising, Germany; daniela.fleckenstein@ivv.fraunhofer.de (D.F.); stephanie.mittermaier@ivv.fraunhofer.de (S.B.-M.); 4 Institute of Food Technology, Hochschule Weihenstephan-Triesdorf, 85354 Freising, Germany; marina.gloning@student.hswt.de; 5 Department of Medicine, Kantonsspital Winterthur, 8400 Winterthur, Switzerland

**Keywords:** malnutrition, nursing home, individualised intervention, enable-cluster, texture-modified diet, oral nutritional supplement

## Abstract

Dietary intake and requirements in nursing home (NH) residents vary individually, but concepts for individualised interventions are currently lacking. Therefore, we present an individualised modular nutritional intervention concept for NH residents with (risk of) malnutrition and describe its application and acceptability. Three enrichment modules—a sweet and a savoury protein cream (40 g, 125 kcal, 10 g protein) and a protein-energy drink (250 mL, 220 kcal, 22 g protein)—were offered to residents of two German NHs single or in combination in five levels of enrichment from level 0 (no enrichment) to 4 (all enrichment modules) to compensate for individual energy and protein deficiencies. Residents with chewing and/or swallowing difficulties received reshaped instead of usual texture-modified meals. The intervention concept was applied to 55 residents (Mean age of 84 ± 8 years, 76.0% female, 25.5% malnutrition). Despite (risk of) malnutrition, 18.2% received no enrichment (level 0). Level 1 was allocated to 10.9%, level 2 to 27.3%, level 3 to 20.0% and level 4 to 23.6% of the residents. 32.7% received reshaped texture-modified meals (RTMM). Participants consuming RTMM were more often assigned to level 4 than residents receiving usual meals (38.8% vs 16.2%). We proposed and successfully applied an individualised modular nutritional intervention concept to NH residents with (risk of) malnutrition. In the next step, the effects of the concept and its transferability to other NHs need to be demonstrated.

## 1. Introduction

Nursing home (NH) residents are at risk population for malnutrition [1]. In German-speaking countries 9–38% of NH residents suffer from malnutrition, and in addition 42–71% are at risk of malnutrition [2]. Negative consequences of malnutrition are multifaceted including functional limitations, reduced quality of life and premature death [3,4,5,6].

Research in the field of prevention and treatment of malnutrition in older adults has mainly focused on the effects of single, standardised interventions, mainly oral nutritional supplements [7,8] and enrichment of meals [9]. As malnutrition is a multifaceted problem, current guidelines recommend that interventions should be more individualised and comprehensive [10]. The rationale for individualised interventions is based on the heterogeneity of older adults in terms of health, functional and cognitive status, preferences, nutritional habits and problems, needs and goals.

Few studies have used individualised approaches to tackle malnutrition in older adults by combining various strategies to meet the nutritional needs of the participants. Randomised controlled trials (RCT) have shown the efficacy of individualised nutritional interventions in improving energy and protein intake as well as body weight in older hospital patients [11,12,13]. Efforts have also been taken in NHs, but the effects of individualised nutritional measures were only examined within multidisciplinary interventions [14,15].

Little has been reported about the procedure to allocate individualised nutritional intervention strategies in older adults. Bounoure and colleagues recently published a standardised, pragmatic concept to allocate individualised treatments to medical inpatients with (risk of) malnutrition [16]. This approach suggests how to identify malnourished or at-risk patients, how to set targets for intake of energy, protein and micronutrients as well as how to choose the type of intervention. To the best of the authors’ knowledge, there is no published, structured approach for the allocation of an individualised nutritional intervention to NH residents.

Accordingly, our aim is to present an individualised modular nutritional intervention concept supporting adequate energy and protein intake in NH residents with malnutrition or risk of malnutrition and to describe application and acceptability of the concept.

## 2. Materials and Methods

The individualised modular nutritional intervention concept was developed in the context of a pre-post study conducted in two NHs in Nuremberg (Germany) between October 2019 and February 2020.

The study was registered at the German Clinical Trials Register (drks.de DRKS00017584) and conducted in accordance with the Declaration of Helsinki about ethical principles for medical research. Approval was given by the ethics committee (Reference Number: 71_19 B) of the Friedrich-Alexander-Universität Erlangen-Nürnberg. Written informed consent was obtained from all participants or their legal representatives.

### 2.1. Intervention Concept

The individualised nutritional intervention was based on the usual catering concept of the NHs and consisted of intervention modules developed in preceding *enable* projects, one module to improve the appearance of texture-modified meals and three modules to provide additional energy and protein (enrichment modules) [17,18].

#### 2.1.1. Usual Care Concept

Both NHs received meals from the same central kitchen. Three main meals (breakfast, lunch, and dinner) and two snacks (in the afternoon and in the late evening) were offered per day during the whole study. Breakfast and dinner were distributed in boxes to the nursing wards. Lunch was delivered to the wards readily prepared on trays in trolleys. At lunch, residents could choose from three menu lines (one vegetarian). Menu lines 1 and 2 were offered in pureed form for residents with chewing and/or swallowing difficulties. Pastries were delivered with lunch and served as an afternoon snack.

#### 2.1.2. Intervention Modules

##### Reshaped texture-modified meals

Pureed meat and vegetables for lunch as well as bread for breakfast and dinner were reshaped using texturisers and silicone moulds. The reshaped diet was developed by the Institute of Food Technology (Weihenstephan-Triesdorf University of Applied Sciences) in close cooperation with the Institute for Biomedicine of Aging (Friedrich-Alexander Universität Erlangen-Nürnberg), following experiences from a previous study [17]. The reshaped meals were produced in the central kitchen of the NHs and offered to participants consuming pureed diet at baseline.

##### Enrichment Modules

A sweet and a savoury protein cream, based on whipped cream and whey protein powder, were offered. To produce the sweet variant, powdered sugar, vanilla sugar and cinnamon were added, and to obtain the savoury variant maltodextrin and spices were added. One portion (40 g) contained 125 kcal and 10 g of whey protein. The protein creams were produced every day in the kitchen of the NHs. Creams were pre-portioned and distributed on the lunch tray of the respective study participants. Nurses were instructed to add the savoury protein cream to the soup or main dish and to serve the sweet protein cream together with the dessert, with the cake as afternoon snack or separately in pure form.

A protein-energy drink was delivered in a 250 mL ready-to drink preparation with orange-mango flavour and contained 220 kcal and 22 g of protein (including 20 g whey protein). The drink was produced for this study by Fraunhofer IVV (Freising, Germany). In daily routine, nursing staff distributed the drink to the respective study participants. The drink could be consumed at once or in several portions spread throughout the day.

#### 2.1.3. Enrichment Levels

Enrichment modules were offered single or in combination to compensate for different extents of energy and protein deficiency. For this purpose, deficiencies were categorised according to the energy and protein content of single or combined enrichment modules following five levels (see Table 1). Energy and protein deficiencies were calculated by subtracting energy and protein intake from energy and protein requirements.

Energy and protein intake was assessed using weighing records on three consecutive days before the beginning of the intervention period.

The calculation of energy and protein intake was based on recipes from the NH kitchen as well as from nutritional information of packaged products. For unpackaged products without nutritional information, the German nutrition database (BLS 3.02, Karlsruhe, Germany) was used. Nutritional analysis of dietary intake was performed with EbisPro 2016 (Willstätt-Legelshurst, Germany).

Energy requirements were estimated by multiplying calculated resting energy expenditure according to Müller et al. (based on body weight, age and sex) [19] by physical activity level (1.2 for inactive, 1.4 for moderately active, 1.6 for very active residents) [20]. Protein requirements were calculated using 1 g protein per kg body weight as reference [20]. In case of any acute or chronic renal disorder, an estimated value of 0.8 g per kg body weight was used. Calculations were based on adjusted body weights when body mass index (BMI) was <22 kg/m^2^ or >27 kg/m^2^ [21].

In addition to individual energy and protein deficiencies, the following aspects were considered to allocate the appropriate enrichment level (EL): BMI (<22, 22–27, or >27 kg/m^2^), weight objective (maintenance or gain), dietary habits (assessed by interviewing nursing staff about meal frequency, eating quantities, taste preferences and individual preferences and allergies) and expected acceptance (assessed by interviewing nursing staff using standardised questions and based on 3-day weighing records). We assigned ELs in structured individual case discussions within the study team. In case of any uncertainties, nursing staff was consulted.

In general, in case of BMI between 22 and 27 kg/m^2^ and/ or desired weight maintenance and equal energy and protein deficiency levels, the corresponding EL was planned. If energy and protein deficiency levels differed, an intermediate level was used.

In case of low BMI (<22 kg/m^2^) and the desire to gain weight, the EL was upgraded or the higher EL was chosen. In case of high BMI (>27 kg/m^2^) and desired weight maintenance the EL was downgraded or the lower EL was targeted.

For residents allocated to EL 2, individual dietary habits and existing nutritional therapy (i.e., oral nutritional supplements or enrichment with maltodextrin) were considered to decide about offering the combination of both protein creams or the protein-energy drink.

### 2.2. Application of the Intervention Concept

#### 2.2.1. Participants’ Recruitment and Characteristics

The intervention was applied to NH residents with (risk of) malnutrition. All residents living permanently in two municipal NHs were screened by research associates supported by nursing staff and using information from care records.

Exclusion criteria were enteral or parenteral nutrition, acute illness, terminal stage of life, age <65 years and BMI ≥30 kg/m^2^. Inclusion criteria were being malnourished or at risk of malnutrition. Malnutrition was defined according to Mini Nutritional Assessment-Short Form (MNA-SF, 7 points or lower [22]). The risk of malnutrition was identified by the following two approaches: Either, by MNA-SF 8–11 points and a reduced score in at least one of the nutrition-related MNA-SF questions (i.e., questions regarding decreased food intake (A), unintentional weight loss (B), psychological stress or acute disease (D), and BMI (F)); or by receiving a texture-modified diet and a reduced score in one of the nutrition-related MNA-SF questions.

Participants’ characteristics were assessed by research associates. Sex, age, chronic diseases, body weight and body height were extracted from care records. Physical activity level (PAL) and dementia (subjective assessment of no, mild or severe dementia) were assessed by interviewing nursing staff using standardised questionnaires. Body weight objective (maintenance, gain), expressed by residents, nurses or research associates, was documented.

#### 2.2.2. Information and Training of Staff

Before the start of the intervention, information events were offered in order to inform nursing staff about the aim and procedures of the study. Kitchen staff was informed accordingly and trained by researchers with practical experience. Production and distribution processes for the intervention were explained in detail and tested before the intervention started.

### 2.3. Acceptability of the Intervention Concept

Regarding acceptability of the concept we describe “how the […] targeted individuals and those involved in implementing programs react to the intervention” (p. 3, [23]).

#### 2.3.1. Acceptability by Residents

Assessment of acceptability by residents included tolerance and compliance.

Regarding tolerance, acute events (gastrointestinal complaints, including nausea/vomiting, diarrhoea and hospital stays) that occurred during the intervention were documented continuously by nursing staff and transferred by research associates into study documentation.

Enrichment was modified or terminated in case of rejection or poor acceptance (according to nurses’ subjective assessment) or health or nutrition-related problems (e.g., gastrointestinal complaints, or severe reduction of habitual food intake) reported by the nursing staff.

Compliance with the enrichment modules and levels was assessed at the beginning and at the end of the six-week intervention period by 3-day weighing records. Offered and consumed amounts of enrichment modules were recorded by research associates.

#### 2.3.2. Acceptability by Staff

Acceptability by staff was assessed within six weeks after the study. Four employees (registered nurses, nursing aides or social care workers) from each of the nine participating nursing wards were asked to evaluate the intervention in terms of usefulness (“I think the protein-energy drink/protein creams/new pureed meals was/were useful”), their practical implementation (“All in all, I was able to integrate administration of the protein-energy drink/protein creams/new pureed meals into my daily routine.”) and their long-term implementation (“All in all, I would support the long-term implementation of the interventions.”). Answers were assessed in written form by using a standardised questionnaire (Likert scale 0–5, “fully disagree” to “fully agree”).

### 2.4. Statistics

Statistical analysis was performed using IBM SPSS Statistics for Windows, version 26.0 (IBM Corp., Armonk, NY, USA).

Categorical variables are presented as n (%). Continuous variables in case of normal distribution are described as mean and standard deviation (SD) and in case of non-normal distribution as median and interquartile range (IQR). Normal distribution was tested using statistical tests (Shapiro–Wilk test) and graphical methods (Histogram). Individual compliance of the intervention modules was expressed as the mean percentage of the consumed compared to the offered amount during the three days. Differences in compliance data between the first and the last week of intervention were tested using Friedman analysis of variance (ANOVA) and differences between the EL using Kruskal–Wallis Test.

## 3. Results

### 3.1. Participants

**Figure 1:**
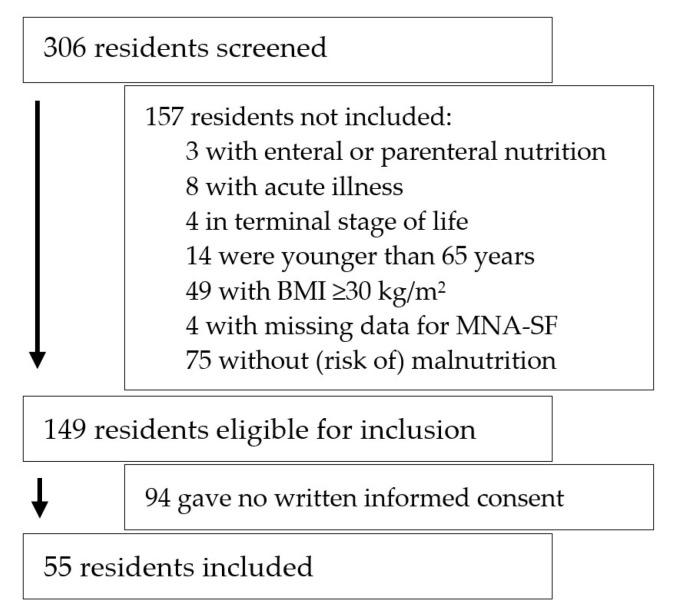
Flow chart of recruitment process. BMI, body mass index; MNA-SF, mini nutritional assessment-short form.

Of the 306 screened residents, 149 were eligible and asked for written informed consent. The intervention concept was applied to 55 residents (Figure 1).

Residents were 84 ± 8 years of age and had a mean BMI of 23 ± 4 kg/m^2^, BMI was <22 kg/m^2^ in 24 and >27 kg/m^2^ in 10 participants. Eighteen residents (32.7%) received texture-modified meals. 76.0% were female, 54.5% severely and 30.9% mildly demented. Fourteen residents (25.5%) were malnourished and 41 (74.5%) were at risk of malnutrition.

### 3.2. Intervention Concept

Median energy deficiency (ED) was 236 (IQR 14-446) kcal per day. Thirteen participants (23.6%) had no energy deficiency (see Table 2), and maximum ED was 1279 kcal/day. Median protein deficiency (PD) was 17 (IQR 9-25) g per day. Nine persons (16.4%) had no PD, and PD was >30 g/day (level 4) in eight residents (14.5%). ED and PD differed by one level in 22 (40.0%) and by more than one level in 13 (23.6%) residents.

Additional consideration of BMI, weight objective, dietary habits and expected compliance resulted in the following assignment of ELs. Ten residents were allocated to an EL according to their deficiency level, because their objective was to maintain weight and they had similar energy and protein deficiencies. We upgraded or chose the higher EL in 22 residents with a BMI <22 kg/m^2^ or 22–27 kg/m^2^ where the objective was to gain weight. We downgraded, chose the lower EL in 19 participants, because the objective was to maintain weight (BMI 22–27 kg/m^2^ or >27 kg/m^2^). Four residents received an intermediate EL as their energy and protein deficiency differed by more than one level.

Overall, 18.2% of the participants received no enrichment (Level 0). Level 2 was assigned most frequently (see Table 3). Within this level, about half (46.6%) of the residents received the protein-energy drink and the other half (53.3%) the sweet and savoury protein cream. A greater proportion of residents receiving reshaped meals, were assigned to EL 4 compared to those receiving usual meals (38.8% vs 16.2%).

The sweet protein cream was allocated to 38 (69.0%), the savoury protein cream to 21 (38.1%) and the protein-energy drink to 31 (56.4%) residents.

### 3.3. Acceptability by Residents

Eight participants had gastrointestinal complaints while receiving an enrichment. The intervention was terminated in two participants because of these complaints. In four residents the intervention was interrupted for one week because of nausea/vomiting (*n* = 1), diarrhoea (*n* = 2) or the combination of both (*n* = 1), and then slowly resumed. In two of these residents the intervention was modified from EL 1 to EL 2. Two residents had gastrointestinal complaints that did not affect the intervention.

Four participants deceased before receiving any intervention. One participant had a hospital stay for more than one week and was therefore excluded from the study. Of the remaining 50 residents, 35 received the sweet protein cream, 19 the savoury protein cream and 27 the protein-energy drink in week 1 of the intervention.

Median compliance of the sweet protein cream, the savoury protein cream and the protein-energy drink was 73% (36–88%), 48% (23–64%) and 96% (51–99%) in week 1 and 57% (26–82%), 44% (0–84%) and 74% (42–97%) in week 6, respectively. Compliance did not differ between the first and the last week of intervention (sweet protein cream: *p* = 0.209, savoury protein cream: *p* = 0.617, protein-energy drink: *p* = 0.102).

Compliance within each EL slightly decreased from week 1 to week 6, except for EL1, although not statistically significant (Table 4). Compliance was lowest in EL 4 where residents consumed only about half of the offered amount.

### 3.4. Acceptability by Staff

Thirty-six staff members (23 registered nurses, 10 nursing aides, 3 social care workers) from nine wards participated in the evaluation. Median length of their employment in the respective nursing home was six years (IQR 3-19).

The protein-energy-drink was perceived useful by 44%, the protein creams by 25% and the reshaped texture-modified meals by 50% of the staff members (“rather/fully agree”). About half (47% for protein creams, 56% for the protein-energy drink, 56% for the reshaped texture-modified meals) stated that they were able to integrate the intervention modules into their daily routine. Overall, 44% of the staff members would support long-term implementation of the intervention modules (“rather/fully agree”), in contrast 11% fully disagreed to do so.

## 4. Discussion

In this study, we proposed and successfully applied an individualised modular nutritional intervention concept for nursing home residents with malnutrition or at risk of malnutrition. By explaining the process of application and the results of this process, we want to enable reproducibility and potential adaption in future research.

Various studies examined the effects of standardised nutritional interventions in nursing homes [24]. However, as requirements and dietary intakes vary between residents it seems reasonable to adjust nutritional interventions in order to allow adequate nutritional intake [10]. Accordingly, we adapted the energy and protein supply for participants by using single or combined enrichment modules that were allocated in structured case discussions, based on calculated energy and protein deficiencies, BMI, weight objective and dietary habits. Additionally, as chewing and/ or swallowing difficulties are a widespread risk factor for malnutrition [1], we optically optimised texture-modified meals by reshaping to enhance enjoyment.

As a basis for participant inclusion, we used the MNA-SF as a validated and widely used malnutrition screening tool in hospitals and residential-care [10,22,25,26]. To include residents because of their nutritional problems and not because of impaired mobility or neuropsychological problems, we added the requirement of limitations in at least one of the nutrition-related criteria of MNA-SF. Although we restricted our inclusion criteria by this procedure to those with nutritional problems, almost one in five participants had no calculated deficiency in energy and/or protein intake and thus did not receive an enrichment. These residents had higher BMI, were younger and tended to have a better functional status in comparison to those receiving an enrichment. No other differences were found (see Table S1). These findings clearly indicate the relevance of an assessment of energy and nutrient intake after screening for malnutrition and before implementing nutritional interventions.

To tackle malnutrition, residents’ nutritional problems and needs would ideally be evaluated within a multidisciplinary team to derive a completely individual nutritional therapy. Since time, money and personnel resources are scarce in nursing homes [27], this individualisation is only possible to a limited extent. Therefore, we used a modular structured intervention, which enables individualisation of energy and protein supply as well as implementation into organisational structures in a practical manner.

We used different enrichment modules and levels to compensate for different degrees of energy and protein deficiencies. In addition, the modules’ taste was prioritised. The protein-energy drink was optimised according to flavour preferences of older people and received positive feedback in pre-tests [18] and the protein creams were offered in two variants to address different taste preferences and to allow them to be used in different meals. Furthermore, we used whey as a protein source because it is considered to be particularly valuable for preserving muscle mass in older adults [28].

Previous research revealed that the intake of texture-modified diets usually lack appearance and taste [29] and are accompanied by lower energy and protein intake compared to usual meals [30]. Therefore, we reshaped the texture-modified diet. The combination of reshaping and enrichment of meals led to positive effects on energy and protein intake as well as body weight in a proof-of-concept study [17].

In order to learn about the practicability of our individualised intervention, the production, distribution and acceptance of the reshaped texture-modified meals and the protein creams were pre-tested [17]. Since we were able to implement the reshaped meals and protein creams in different institutions and catering systems (with bulk foods [17] and tray-based), our assumption of practicability was verified. Our protein-energy drink could be distributed on the nursing wards in the daily routine like conventional oral nutritional supplements, and production and distribution of the protein creams was well practical after initial training.

Recent research with an innovative enriched brioche showed good acceptability and improved energy intake of nursing home residents with malnutrition or at risk receiving intervention compared to the group receiving oral nutritional supplements [31]. These results demonstrate the successful use of innovative products to treat malnutrition. In contrast, the primary focus of the present work was on a new concept, its application, and acceptability. In a next step, its effects on dietary intake and nutritional status will be examined, firstly compared to usual care. Comparison to other specific interventions, e.g., oral nutritional supplements, may be an interesting and relevant topic for future research.

Individualised nutritional interventions are usually defined through individual counselling, for example by a dietician [11,14,32]. In order to make the allocation of enrichment modules comprehensible and replicable, we defined specific criteria and structured the decision process. Energy and protein deficiencies, BMI and weight objective were used as the main criteria for intervention allocation. The enrichment concept was based on energy and protein deficiencies, because a nutritional intervention should support adequate dietary intake based on individually varying requirements. BMI and weight objective were used because they provide objective information on the nutritional status of a person and they are relevant factors regarding requirements and the amount of food that should be offered [10,33]. The weight objective is additionally important because it should take the individual weight into account. In addition, to support acceptance and thus effectiveness of the enrichment, we included individual dietary habits and preferences.

The success of an intervention is largely dependent on its acceptability by participants. We did not find any severe complications in tolerance linked to our intervention. The risk of adverse events caused by the intervention was expected to be low because the modules consisted of usual food components. To ensure safety, allergies and general gastrointestinal complaints were recorded and considered in the application of the intervention. Additionally, adverse events were documented, a possible link to the intervention was discussed, and the intervention was adjusted if necessary. One-fifth (20%) of the residents receiving an enrichment had gastrointestinal complaints. To avoid further complications, the intervention was modified or terminated in 15%. In a study using oral nutritional supplements in NH residents, gastrointestinal complaints were reported in 33% [34].

Overall, compliance with our intervention modules was good, as median intake ranged between 44% and 96% and was comparable to the median compliance of oral supplements reported in intervention studies with nursing home residents, which for instance were 56% [35] and 73% [34]. Established intervention forms such as oral nutritional supplements, like our protein-energy drink, seem to be better accepted than the innovative protein creams, especially in the savoury variant. The rather low compliance of the savoury protein cream could be explained by the fact that people generally tend to consume more sweet foods with increasing age [36]. When interpreting the results on compliance with our modules or within EL, the small group size and large variance should be taken into account by drawing only moderate conclusions. Median compliance of the protein-energy drink decreased from 96% in week 1 to 74% in week 6, however variance was large. This decrease may be explained by several factors, including declining motivation of the staff administering the drink, or by residents experiencing taste fatigue [37].

Interestingly, compliance with the enrichment modules was also good in the highly represented subgroup of residents with severe (68% (57–84%), *n* = 30) and mild dementia (64% (8–83%), *n* = 17; without dementia: 43% (38–44%), *n* = 8; data from week 6; data not shown), although lower compliance could have been expected because of memory problems, adverse eating behaviour and possibly reduced appetite [8,10,38].

Some aspects concerning compliance are additionally noticeable. Interestingly, the highest level of enrichment (EL 4) had the lowest compliance, which might be a result of the higher offered amount of enrichment and the rather poor compliance of the savoury protein cream. Furthermore, we observed that compliance did not change between the first and the last week of intervention. We see these results in line with a systematic review on compliance to oral supplements, in which compliance was not correlated with the duration of the intervention period [39].

Acceptability by staff was overall good and better than reported in a study investigating the attitude of nursing staff towards malnutrition care in nursing homes, where only 39% had a positive attitude towards nutritional care [40]. Nursing staff, comparable to residents, accepted the protein-energy drink better than the protein creams. Overall, we consider the implementation of the intervention modules practicable, as about half of the staff reported being able to integrate the modules into their daily routine and 44% would support their long-term implementation.

Our intervention concept has limitations that are worth mentioning. First, we used 3-day weighing records to assess energy and protein intake. However, this approach is time-consuming and thus limits direct implementation of our concept in daily routines. For clinical practice, when no dietitian is available, a simple and quick but nevertheless reliable instrument for the assessment of energy and protein intake and calculation of deficiencies is needed. An option to increase practicality is the use of plate diagrams which are reliable if the offered amounts are known [41]. However, in this area, further studies are required to demonstrate the validity of these simple methods for different catering systems. Second, various assessments in our study are based on subjective evaluations by nurses (e.g., physical activity level, weight objective, dietary habits). The quality of this data might be influenced by staff ratio, time and communication [27] and should therefore be used and interpreted with care. However, research indicates that nurses are attentive to key aspects of food intake and body weight [42,43]. Third, individualisation of our intervention was only possible to a limited extent. We did not include micro- or additional macronutrients in the study concept, as further assessments and intervention modules would have increased the complexity. Addressing micronutrients would be another important step as inadequate intakes are frequently reported in nursing home residents [44]. In addition, energy and protein supplementation could not be separated, because protein always provides energy and also to keep the concept feasible. To avoid oversupply of energy, we excluded residents with a BMI ≥30 kg/m^2^.

## 5. Conclusions

We developed an individualised modular nutritional intervention concept for nursing home residents with (risk of) malnutrition and observed good applicability and acceptability. The concept was applied in a transparent manner in case discussions by considering individual energy and protein deficiencies, residents’ BMI, weight objective and dietary habits. Application of the implementation was supported by the modular structure and the focus on energy and protein supply. In the next step, the effects of the concept and its transferability into catering and nursing systems of other nursing homes need to be investigated. This paper addresses existing problems of individualised intervention in the nursing home sector and, by describing the concept and its acceptability, is intended to facilitate future studies.

## Figures and Tables

**Table 1:** Energy and protein deficiency, enrichment modules and their energy and protein content in 5 levels of enrichment.

Level	Deficiency		Enrichment		Allocated Modules
	Energy [kcal]	Protein [g]	Energy [kcal]	Protein [g]	Protein Creams (40 g per Portion)
					Protein-Energy Drink (250 mL per Portion)
0	<0	<0	0	0	-
1	0–150	0–10	+125	+10	Sweet protein cream
2	151–250	11–20	+220–250	+20–22	(Sweet and savoury protein cream) or protein-energy drink
3	251–350	21–30	+345	+32	Sweet protein cream and protein-energy drink
4	>350	>30	+470	+42	Sweet and savoury protein cream and protein-energy drink

**Table 2:** Number and percentage of residents in energy and protein deficiency levels (*n* = 55).

Level of Deficiency	Energy Deficiency		Protein Deficiency	
	*n*	%	*n*	%
0	13	23.6	9	16.4
1	8	14.5	5	9.1
2	7	12.7	18	32.7
3	4	7.3	15	27.3
4	23	41.8	8	14.5

**Table 3:** Number of residents in enrichment levels stratified by reshaped texture-modified meals.

Level of Enrichment	Reshaped Texture-Modified Meals				Total	
	No	(*n* = 37)	Yes	(*n* = 18)	(*n* = 55)	
	*n*	%	*n*	%	*n*	%
0	7	18.9	3	16.6	10	18.2
1	4	10.8	2	11.1	6	10.9
2	10	27.0	5	27.7	15	27.3
3	10	27.0	1	5.6	11	20.0
4	6	16.2	7	38.8	13	23.6

**Table 4:** Median intake (%) of the offered enrichment levels in week 1 and 6 of the intervention (*n* = 50).

Level of Enrichment		Week 1		Week 6	*p*-Value *
	*n*	Median (IQR)%	*n*	Median (IQR)%	
0	10	-	10	-	-
1	6	78 (36–88)	4	82 (40–85)	0.564
2	12	83 (74–99)	14	61 (48–78)	0.109
3	10	77 (49–91)	10	68 (44–73)	0.527
4	12	53 (24–69)	12	45 (15–79)	0.527

* Friedman analysis of variance. EL, enrichment level; IQR, interquartile range.

## Data Availability

The data presented in this study might be available on request from the corresponding author. The data are not publicly available due to residents or legal representatives not giving full consent.

## Supplementary Materials

The following are available online at https://www.mdpi.com/2308-3417/6/1/2/s1, Table S1: Participants’ characteristics in the total sample and stratified by enrichment (*n* = 55).
