# Supplementary material for: An Individualised Nutritional Intervention Concept for Nursing Home Residents with or at Risk of Malnutrition: An enable Study
Source: Geriatrics (Basel). 2020 Dec 26;6(1):2. doi: 10.3390/geriatrics6010002 (PMC7838920; doi:10.3390/geriatrics6010002)
Supplement: Supplementary file 1 [file geriatrics-06-00002-s001.pdf]

Supplement Table 1. Participants' characteristics in the total sample and stratified by enrichment (n = 55)

		Total	Without Enrichment (EL0)	With Enrichment (EL1–4)	
		n = 55	*n* = 10	n = 45	
		n (%)/ median (IQR)/ mean (SD)			p-value
Female gender		42 (76.4%)	7 (70.0%)	35 (77.8%)	0.601 [a]
Age [years]		84 (±8)	79 (±7)	86 (±8)	0.011 [b]
Barthel-Index [points]		35 (5-65)	73 (5-95)	30 (5-55)	0.087 [c]
CFS [points]		7 (6-7)	6 (6-7)	7 (7-7)	0.066 [c]
Dementia	Severe	30 (54.5%)	5 (50.0%)	35 (55.6%)	0.861 [a]
	Mild	17 (30.9%)	3 (30.0%)	14 (31.1%)	
	No	8 (14.5%)	2 (20.0%)	6 (13.3%)	
MNA-SF	Malnourished	14 (25.5%)	2 (20.0%)	12 (26.7%)	0.662 [a]
	Risk of MN	41 (74.5%)	8 (80.0%)	33 (73.3%)	
Texture-modified meals		18 (32.7%)	3 (30.0%)	15 (33.3%)	0.662 [a]
Body Mass Index [kg/m^2]		23.0 (±3.8)	27.0 (±3.7)	22.1 (±3.22)	<0.001[b]

[a] Chi-squared-test, [b] t-test, [c] Mann-Whitney-U-test. BMI, Body Mass Index; CFS, Clinical Frailty Scale; EL, enrichment level; IQR, interquartile range; MNA-SF, Mini Nutritional Assessment Short Form; MN, Malnutrition; SD, Standard deviation.
